# Chest Pain: The Need to Consider Less Frequent Diagnosis

**DOI:** 10.1155/2016/4294780

**Published:** 2016-02-29

**Authors:** Pedro Magalhães, Anabela Morais, Sofia Carvalho, Joana Cunha, Ana R. Lima, J. Ilídio Moreira, Trigo Faria

**Affiliations:** ^1^Serviço de Cardiologia, Centro Hospitalar de Trás-os-Montes e Alto Douro, 5000-508 Vila Real, Portugal; ^2^Serviço de Medicina Interna, Centro Hospitalar de Trás-os-Montes e Alto Douro, 5000-508 Vila Real, Portugal

## Abstract

Chest pain is one of the most frequent patient's complaints. The commonest underlying causes are well known, but, sometimes, in some clinical scenarios, it is necessary to consider other diagnoses. We report a case of a 68-year-old Caucasian male, chronically hypertensive, who complained of recurrent episodes of chest pain and fever with elevated acute phase reactants. The first investigation was negative for some of the most likely diagnosis and he quickly improved with anti-inflammatory drugs. Over a few months, his symptoms continued to recur periodically, his hypertension was aggravated, and he developed headaches and lower limbs claudication. After a temporal artery biopsy that was negative for vasculitis, he underwent a positron emission tomography suggestive of Takayasu Arteritis. Takayasu Arteritis is a rare chronic granulomatous vasculitis of the aorta and its first-order branches affecting mostly females up to 50 years old. Chest pain is experienced by >40% of the patients and results from the inflammation of the aorta, pulmonary artery, or coronaries.

## 1. Introduction

Chest pain is one of the most frequent symptoms driving patients to a physician's practice or to the hospital's emergency department. Although the prevalence of each aetiology differs according to the clinical setting, the underlying causes of chest pain are more commonly due to gastrointestinal, cardiac, chest wall/musculoskeletal, respiratory/pulmonary, and psychiatric disorders [1–3]. Nevertheless, in some clinical scenarios and after the exclusion of these aetiologies, it is important to consider other less common causes. We are presenting a clinical case that is representative of such investigation.

## 2. Case Report

A 68-year-old Caucasian male presented to the emergency department (ER) with a 6-day history of intense retrosternal pain, radiating to the dorsum and left arm, plus fever. He also had a history of high blood pressure (HBP), medicated with olmesartan 20 mg id and headaches. On admission, he was hypertensive (162/81 mmHg) and febrile (38.2°C) without any other abnormalities at physical examination. His chest X-ray and electrocardiogram were normal. Blood analysis revealed elevated erythrocyte sedimentation rate (ESR, 81 mm) and C-reactive protein (CRP, 9.2 mg/dL) and normal myocardial necrosis markers. A transthoracic echocardiogram (TTE) was performed showing a mild pericardial thickening. Acute pericarditis was assumed and the patient was discharged with a nonsteroidal anti-inflammatory drug (NSAID).

The patient returned to the ER 2 days later with the same complaints. He maintained high acute phase reactants (APR) (ESR 82 mm, CRP 11.1 mg/dL), without other physical, laboratorial, or radiological changes, and he was then admitted to the Internal Medicine Service for further investigation. Through several virologic and serologic markers, we excluded tuberculosis, syphilis, infection by* Brucella*,* Rickettsia*,* Salmonella*, hepatitis B and hepatitis C virus, HIV, influenza, parainfluenza, adenovirus, and Coxsackie A and Coxsackie B. We did not isolate any microorganisms in blood or urine cultures. Myocardial necrosis markers and d-dimers did not rise. Autoantibody tests (antinuclear, anti-dsDNA, anti-neutrophil cytoplasmic, antiphospholipid, and anti-citrulline antibodies and rheumatoid factor) were proven negative and angiotensin converting enzyme levels were normal. He repeated TTE which was normal and underwent an abdominal and renal ultrasound that displayed no significant alterations. When under NSAID treatment, his symptoms ceased, the APR levels diminished, and he did not show any other analytical abnormalities. He was discharged 5 days later and referred to the Internal Medicine's ambulatory for further workup.

In the meantime, the patient developed recurrent episodes of chest pain, claudication of the lower limbs, headaches, and HBP aggravation which imposed prescription reinforcement by the general practitioner (olmesartan + hydrochlorothiazide).

**Figure 1:**
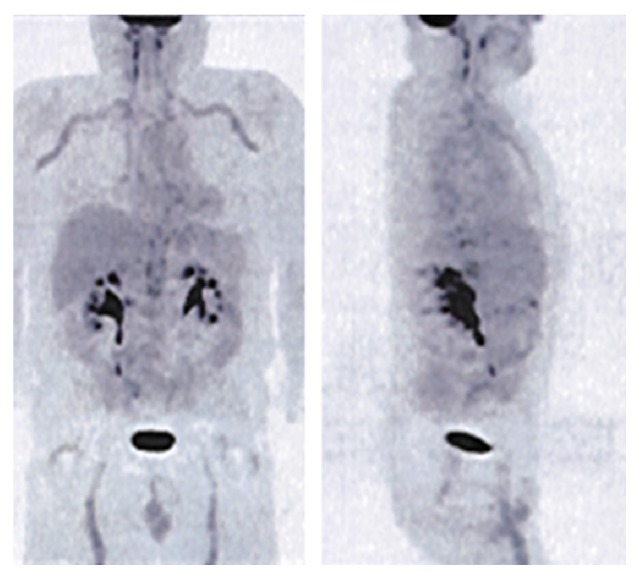
PET scan showing increased uptake of 18F-FDG at the subclavian, carotid, humeral, vertebral, and femoral arteries and less intensively at the ascending and descending aorta, a pattern suggestive of TA. PET: positron emission tomography. 18F-FDG: 18F-fluorodeoxyglucose. TA: Takayasu Arteritis.

**Figure 2:**
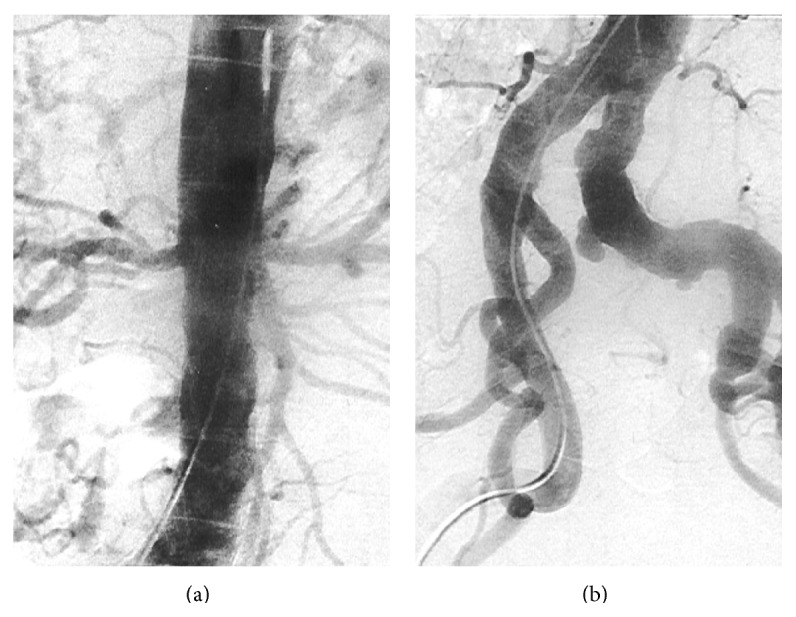
Arteriography showing diffuse areas of narrowing and dilation of the abdominal aorta (a) and significant dilation of the left common iliac artery (b).

Six months later, he returned to the ER with a 12-day history of fever and severe headaches. Once again laboratory analysis showed not only elevated APR (ESR 120 mm, CRP 11.4 mg/dL), but also normochromic normocytic anaemia (haemoglobin 11.6 g/dL) and hypoalbuminemia (albumin 3.0 g/dL). The chest X-ray, TTE, and cerebral computed tomography were normal. The patient was admitted to the Internal Medicine Service for further investigation and underwent a temporal artery biopsy, which was negative for vasculitis. We then decided to perform a positron emission tomography (PET) that showed an increased uptake of 18F-fluorodeoxyglucose (FDG) at the subclavian, carotid, humeral, vertebral, and femoral arteries and less intensively at the ascending and descending aorta, suggestive of Takayasu Arteritis (TA) (Figure 1). An arteriography was also performed revealing diffuse areas of narrowing and dilation at the aorta and main branches (Figure 2). Oral prednisolone (1 mg/kg) was prescribed with symptoms resolution and the patient was discharged under the same therapeutics.

**Figure 3:**
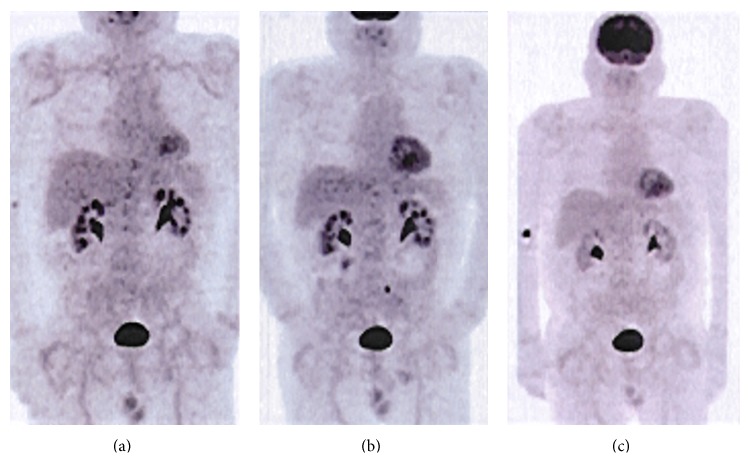
Sequential PET scan images. (a) One-year follow-up: medium intensity uptake of 18F-FDG at the subclavian, carotid, and femoral arteries. (b) Two-year follow-up: medium intensity uptake of 18F-FDG at the femoral and right external iliac. (c) Three-year follow-up: absence of 18F-FDG uptake. PET: positron emission tomography. 18F-FDG: 18F-fluorodeoxyglucose.

The patient was reassessed on an outpatient basis 1 month later, free of symptoms, with stabilized BP and normalization of the APR and haemoglobin. By the end of the first year of treatment, he repeated PET that showed radiologic improvement but still metabolic activity of 18F-FDG (Figure 3(a)) and he was then started on methotrexate 15 mg/week. The two-year follow-up PET revealed additional radiologic improvement allowing prednisolone suspension and reduction of the methotrexate dosage (7.5 mg/week) (Figure 3(b)). The three-year follow-up PET did not show any 18F-FDG uptake (Figure 3(c)). This time around, the patient was hospitalized for uncomplicated influenza pneumonia. Methotrexate was suspended and it was decided not to reinitiate it, taking under consideration the last PET result. Since then, without any prednisolone or methotrexate, he has remained symptom free, with mild APR elevation and without any disease associated complications.

## 3. Discussion

TA is a relatively rare chronic idiopathic granulomatous large vessel vasculitis (LVV) affecting the aorta and its first-order branches, with an estimated prevalence of 2.6/1,000,000 persons in the United States and 1.26/1,000,000 persons in northern Europe [4, 5]. It tends to affect patients up to 50 years old with female gender predominance (80–90%). The age of onset is usually between 10 and 40 years. Although there is a considerable variability on disease expression, the initial vascular lesion often starts in the left subclavian artery and subsequently spreads to involve the left common carotid, left vertebral, brachiocephalic, right subclavian, right vertebral, and right common carotid arteries. Thoracic aorta is commonly affected, whereas abdominal aorta and pulmonary arteries are involved in 50% of the patients [4].

Symptoms resulting from systemic inflammation, such as fever, weight loss, fatigue, malaise, arthralgias, or myalgias, are common in the early stage of the disease and may represent the systemic effects of cytokines [4].

Vascular symptoms and signs are rare at presentation and reflect the affected arterial territories. Chest pain is experienced by >40% of the patients and results from the inflammation at the aortic arch or root level (affected in 35% of the patients), pulmonary artery (10–40%), or the coronaries (<10%) [6, 7]. Headaches are a consequence of the carotid and vertebral arteries involvement with decreased cerebral blood flow affecting 45% of the patients [7, 8]. Lower limb claudication occurs in 18–30% of the patients and reflects disease of the iliac [7, 9]. Hypertension is developed in more than a half of the cases due to the narrowing of the renal artery or narrowing and decreased elasticity of the aorta and branches [5].

Interestingly, far from the commonest scenario, our patient is a 68-year-old male. He shows both systemic and vascular symptoms. His chest pain was most likely related to the involvement of the ascending aorta and his multiple and different symptoms reflect the disseminated character of his disease.

Laboratory changes reflect the inflammatory process and include elevated ESR and CRP, normochromic normocytic anemia, and hypoalbuminemia [6]. Nevertheless, about 25–50% of the patients have a normal acute phase response even in the presence of active disease, which also makes ESR and CRP unreliable parameters to monitor disease's activity if used as unique criteria [4, 9]. Some autoantibodies, such as antiendothelial cell antibodies, have been implicated in the pathogenesis of the disease, but other autoantibodies associated with other forms of vascular disease, including antinuclear, antineutrophil cytoplasmic, anti-DNA, and antiphospholipid autoantibodies, are not found in TA [10]. Our patient had negative antinuclear, antineutrophil cytoplasmic, anti-DNA, and antiphospholipid antibodies as well.

The diagnosis of TA is usually considered upon suggestive clinical features and imaging of the arterial tree by arteriography, magnetic resonance imaging (MRI), or computed tomography (CT) that demonstrate the characteristic pattern of irregular vessel walls, stenosis, poststenotic dilation, aneurysm formation, occlusion, and evidence of increased collateral circulation [6]. MRI and CT also allow the evaluation of the vessels wall thickness. PET is another frequently used technique that detects arterial inflammation based on the vascular FDG uptake [11]. Considerable controversies concerning the modality that is best for diagnosis and follow-up exist, since there are no published comparative studies to guide us regarding the optimal imaging modality. Also, there is no consensus on how often these imaging studies should be repeated. In general, they are recommended when a relapse is suspected or, when asymptomatic, at least annually to exclude disease progression or worsening due to mechanical factors [4, 11]. Noninvasive imaging methods are essential for monitoring disease activity and response to treatment because, as discussed earlier, there is a lack of positive correlation between disease activity and the rise of APR in a very significant proportion of patients [12].

Several disorders, including many forms of vasculitis, must be distinguished from TA. One of the most important and difficult differential diagnoses is Giant Cell Arteritis (GCA), another granulomatous LVV that characteristically involves one or more branches of the carotid artery, particularly the temporal one, but it can also affect arteries in multiple locations and occurs almost exclusively in individuals older than 40–50 years. In fact, the age of onset of the disease is one of the most discriminatory characteristics between the two pathologies [6, 13].

In this case, considering the patient's initial complaints of chest pain and fever, we first thought of a myopericardial syndrome, but the electrocardiogram, TTE, and myocardial necrosis markers were normal. Although less likely, the hypothesis of an acute coronary syndrome was ruled out for the same reasons. We also considered a parenchymal/vascular pulmonary disease, but the atypical chest pain, normal chest X-ray, and normal leucogram made pneumonia improbable and the patient also had a low clinical likelihood for pulmonary embolism and negative d-dimers. The diagnosis of esophagitis was very unlikely, especially in an immunocompetent patient and no evidence existed to support a chest wall/musculoskeletal origin. During the first hospitalization, we have excluded many infectious aetiologies and the autoantibody tests were negative. The episodic character of the symptoms along with the new ones (headaches and limbs claudication) and the laboratorial changes (elevated APR, normochromic normocytic anaemia, and hypoalbuminemia) raised the suspicion for a systemic disease such as a vasculitis, in particular medium and large vessels one. Considering his age and headaches complaints, we first thought of GCA, and therefore we performed a temporal artery biopsy, which was negative for vasculitis. Once again, because his several and varied symptoms suggested a systemic vasculitis, we then decided to go for a broader test, thence the option for the PET scan that led us to the diagnosis of TA. We opted to keep PET for imaging follow-up and we consider our series of sequential images to be highly illustrative of the important role this image modality might play both for TA diagnosis and for follow-up.

In the presence of active disease, the initial standard treatment of TA is with high doses of prednisolone (1 mg/kg/day) or its equivalents for about a month, which are then gradually tapered until discontinuation. Glucocorticoids induce remission in about 60% of the patients, but relapses do occur in the majority (>50%) during steroid taper. In these cases, or when there is a need to counteract the side effects of steroids, a conventional immunosuppressive agent is added, usually methotrexate (25 mg/week). When these agents or a combination of these agents remain ineffective, or are not tolerated, biologic agents may be tried out [4, 12, 14]. We followed these recommendations, our patient responded fairly well to prednisolone in the acute phase and we were able to achieve excellent control of the disease with a low methotrexate dosage (7.5 mg/week).

TA is considered to be a serious disease with a chronic relapsing-remitting course causative of significant morbidity and disability. The long-term outcome of patients with TA has varied widely between studies, with a 5-year mortality rate ranging from 0 to 35% [4, 6, 9]. A recent cohort study reports an increased mortality compared to the general population (standardized mortality ratio of 3.0) with 5-year, 10-year, and 15-year survival rates of 97%  ±  2%, 97%  ±  2%, and 86%  ±  6%, respectively [7]. Seven years after the diagnosis, our patient is doing well, being free of symptoms, and being without any disease's complications.

We believe this case highlights the importance of considering less common diagnosis when we are dealing with unexplained symptoms, especially after a relatively exhaustive first line investigation. When the clinical context strongly suggests a chronic, persistent, and inflammatory process, autoimmune aetiology must be considered.

## References

[1] Lee T. H., Fauci A. S., Kasper D. L., Hauser S. L., Jameson J. L., Loscalzo J., Longo D. L. (2012). Chest discomfort. *Harrison's Principles of Internal Medicine*.

[2] Sabatine M. S., Cannon C. P., Bonow R. O., Mann D. L., Zipes D. P., Libby P., Braunwald E. (2012). Approach to the patient with chest pain. *Braunwald's Heart Disease: a Textbook of Cardiovascular Disease*.

[3] Erhardt L., Herlitz J., Bossaert L. (2002). Task force on the management of chest pain. *European Heart Journal*.

[4] Chatterjee S., Flamm S. D., Tan C. D., Rodriguez E. R. (2014). Clinical diagnosis and management of large vessel vasculitis: takayasu arteritis. *Current Cardiology Reports*.

[5] Kerr G. S., Hallahan C. W., Giordano J. (1994). Takayasu arteritis. *Annals of Internal Medicine*.

[6] Langford C. A., Fauci A. S., Longo D. L., Fauci A. S., Kasper D. L., Hauser S. L., Jameson J. L., Loscalzo J. (2012). The vasculitis syndromes. *Harrison's Principles of Internal Medicine*.

[7] Schmidt J., Kermani T. A., Bacani A. K. (2013). Diagnostic features, treatment, and outcomes of Takayasu arteritis in a US cohort of 126 patients. *Mayo Clinic Proceedings*.

[8] Rodríguez-Pla A., de Miguel G., López-Contreras J., de Llobet J. M., Llauger J., Díaz C. (1996). Bilateral blindness in Takayasu's disease. *Scandinavian Journal of Rheumatology*.

[9] Forte A. V., Mandell B. F., Bonow R. O., Mann D. L., Zipes D. P., Libby P., Braunwald E. (2012). Rheumatic diseases and the cardiovascular system. *Braunwald's Heart Disease: A Textbook of Cardiovascular Disease*.

[10] Eichhorn J., Sima D., Thiele B. (1996). Anti-endothelial cell antibodies in Takayasu arteritis. *Circulation*.

[11] Chatterjee S., Flamm S. D., Tan C. D., Rodriguez E. R. (2014). Clinical diagnosis and management of large vessel vasculitis: giant cell arteritis. *Current Cardiology Reports*.

[12] Keser G., Direskeneli H., Aksu K. (2014). Management of Takayasu arteritis: a systematic review. *Rheumatology*.

[13] Michel B. A., Arend W. P., Hunder G. G. (1996). Clinical differentiation between giant cell (temporal) arteritis and Takayasu's arteritis. *Journal of Rheumatology*.

[14] Terao C., Yoshifuji H., Mimori T. (2014). Recent advances in Takayasu arteritis. *International Journal of Rheumatic Diseases*.

